# Comprehensive Investigation on Principle Component Large-Scale Wi-Fi Indoor Localization †

**DOI:** 10.3390/s19071678

**Published:** 2019-04-08

**Authors:** Ahmed H. Salamah, Mohamed Tamazin, Maha A. Sharkas, Mohamed Khedr, Mohamed Mahmoud

**Affiliations:** 1 Electrical and Computer Engineering Department, University of Waterloo, Waterloo, ON N2L 3G1, Canada; AhmedHuss.Salamah@aast.edu; 2 Electronics and Communications Engineering Department, Arab Academy for Science, Technology and Maritime Transport (AASTMT), Alexandria, Egypt; msharkas@aast.edu (M.A.S.); khedr@aast.edu (M.K.); drmmaly@aast.edu (M.M.)

**Keywords:** Wi-Fi indoor navigation, machine learning, principal component analysis

## Abstract

The smartphone market is rapidly spreading, coupled with several services and applications. Some of these services require the knowledge of the exact location of their handsets. The Global Positioning System (GPS) suffers from accuracy deterioration and outages in indoor environments. The Wi-Fi Fingerprinting approach has been widely used in indoor positioning systems. In this paper, Principal Component Analysis (PCA) is utilized to improve the performance and to reduce the computation complexity of the Wi-Fi indoor localization systems based on a machine learning approach. The experimental setup and performance of the proposed method were tested in real indoor environments at a large-scale environment of 960 m^2^ to analyze the performance of different machine learning approaches. The results show that the performance of the proposed method outperforms conventional indoor localization techniques based on machine learning techniques.

## 1. Introduction

Over the last decades, ubiquitous positioning has become a significant topic that the research community continues to carry out today. The most popular and reliable solution in the commercial navigation market is using GPS receivers. The GPS suffers from outages and multipath at indoor localization environments. There is a spectacular increasing of indoor localization studies that has been observed in the last decades [1]. However, the complex structure and the dynamic nature of indoor environments pose several challenges to develop accurate indoor positioning systems [2].

Since the Wireless Local Area Network (WLAN) infrastructures are widely implemented in public areas on many indoor environments, the WLAN-based positioning systems have become the most popular low-cost indoor positioning solution. In the literature [3,4], there are two main approaches based on Received Signal Strength Indicator (RSSI) signal level, i.e., the power of received signals from WLAN Access Points (APs) were categorized to model-based (path loss) and model-free (radio map). The model-based approach is based on collecting the RSSI to train the parameters for the predefined propagation models. These techniques assume a prior path loss model for the indoor propagation, which is a logarithmic decay function of the distance from the APs [5]. Due to the randomness of propagation effects after applying path loss propagation model in the indoor environment, the model led to unfeasibility of distance estimation resulting in wrong position [6].

The model-free fingerprinting approach uses the randomness of the propagation effect to distinguish between one position and another. It is widely used in the literature for its robustness and cost-effectiveness to overcome the limitations of the model-based approach [7]. Many difficulties involved in the design of indoor positioning systems are intrinsic to WLAN using fingerprint approach. For example, in a typical WLAN-based positioning system, the computational complexity, battery power, and storage capacity need to be jointly considered, where the computations of the mobile user location take place on a battery-powered device with limited processing capability and limited power supply. Consequently, using algorithms of low computational complexity is required for indoor positioning. The dimension of the RSSI features (number of reachable APs) is one of the most critical factors determining the computational complexity of the whole positioning process. Therefore, choosing a small subset of detectable APs is an intuitive way to reduce the computation load required from the mobile device, while achieving the same level of accuracy. Moreover, WLAN-based positioning systems should be designed to concurrently support a large number of users to be compatible with the huge user base of WLAN. Simple algorithms accelerate the location finding process and hence enable accommodating a more significant number of users.

The ultimate goal of this research is to reduce the required computational complexity cost with enhancing or maintaining the accuracy level of the Wi-Fi indoor localization systems based on machine learning approaches. The research is mainly concerned with four design aspects of indoor positioning systems, which are precision, accuracy, scalability, and complexity. The Principal Component Analysis (PCA) utilized in this research to identify the redundancy structure resulting from multivariate APs and to reduce the duplicated fingerprint and noise between APs.

## 2. Related Work

The Indoor localization systems based on the fingerprint approach can be classified into three categories: The deterministic approach, probabilistic approach, and machine learning approach [8]. The widely known Wi-Fi RADAR system [9] is the first RF-based localization system using the deterministic approach of the WLAN fingerprint. The Wi-Fi RADAR system stores the RSSI fingerprints at grid points in the offline stage, which creates the radio map. The K-nearest neighbor method is used to find the estimated location for the user’s fingerprint.

The probabilistic approach is based on computing the probability of each grid point and estimating the user’s position using the Bayesian inference [10]. This approach is used in the HORUS system, where it achieves higher accuracy in comparison to the Wi-Fi RADAR system that is based on the deterministic approach [11]. The main drawback of the probabilistic approach is that it requires a large number of samples from APs to create a distribution. This increases the time required to build the radio map and requires large storage size.

The COMPASS [12] system is another probabilistic indoor positioning system. This system is developed to consider the attenuation caused by the human body by adding a compass to the system. In the offline stage, the COMPASS system creates multiple radio maps with several selected orientations (typically each 45° or 90°). In the online stage, the user orientation is provided with a digital compass, and only the fingerprints with a similar orientation are utilized to estimate the user’s location. The main disadvantage of the COMPASS system is that it requires a large storage size to save several radio maps with different orientations.

Due to the significant computational cost of the approaches mentioned above, the researchers begin to pay more attention to the machine learning approach (e.g., the artificial neural network [13], Support Vector Machine [14], and Fuzzy Logic [15] to realize the fingerprint positioning. The most important advantage of the machine learning approach is about the real-time ability to infer the user’s coordinates in the on-line phase. Therefore, this paper focuses on the performance of the Wi-Fi indoor localization system based on a machine learning approach.

## 3. Proposed Method

Principal Component Analysis (PCA) [3,16] is a dimensionality reduction method that deals with high dimensionality problems by linearly combining features into a lower dimensional space. PCA uses the knowledge of the training data covariance matrix to decorrelate the features and to project the data in the direction of the most significant variance. Therefore, PCA can be used to choose the most informative APs in any region given its radio map. However, PCA has a significant drawback when used for classification since PCA preserves the features with the maximum variance, but not the most discriminative features. Therefore, PCA dimensionality reduction might be at the cost of degradation in the positioning accuracy, which is against the objective of maintaining high positioning accuracy. Thus, to overcome this issue, some analysis on the number of Principal Components (PCs) will be considered.

The proposed method is based on choosing a subset of features, where the approach replaces the elements with a subset of PCs instead of choosing a subset of APs, which are obtained by a transformation of the measured RSSI. PCA provides an effective transformation such that the entropy and the retained information in the chosen PCs is maximized. The idea of AP selection can be expressed in a simple mathematical form as shown in Equation (1).

(1) $${\left[\begin{array}{c} y_{1}\\ \begin{array}{c} M\\ \begin{array}{c} M\\ y_{U} \end{array} \end{array} \end{array}\right]}_{U\times 1}={\left[\begin{array}{ccc} 1 & 0 & \begin{array}{cc} L & 0 \end{array}\\ 0 & 1 & \begin{array}{cc} L & 0 \end{array}\\ \begin{array}{c} M\\ 0 \end{array} & \begin{array}{c} M\\ L \end{array} & \begin{array}{c} \begin{array}{cc} M & M \end{array}\\ \begin{array}{cc} 1 & 0 \end{array} \end{array} \end{array}\right]}_{U\times M}{\left[\begin{array}{c} x_{1}\\ M\\ \begin{array}{c} M\\ x_{M} \end{array} \end{array}\right]}_{M\times 1},$$

where the vector $X={\left[x_{1},{\;x}_{2},\;K,{\;x}_{M}\right]}^{T}\in R^{M\times 1}$, represents the measurement from available M APs, the matrix $A\in R^{U\times N}$, U≤M, contains the selective weighting describing which APs are chosen, and the vector $Y={\left[y_{1},{\;y}_{2},\;K,{\;y}_{M}\right]}^{T}\in R^{U\times 1}$, represents the measurement from the selected APs. In Equation (2), the first U APs among $\left[x_{1},{\;x}_{2},\;K,{\;x}_{M}\right]$ are chosen and, thus, $\left\{x_{1},{\;x}_{2},\;K,{\;x}_{U}\right\}=\left\{y_{1},{\;y}_{2},\;K,{\;y}_{U}\right\}$. Therefore, the regarded method is used as an individual information source selection approach.

Unlike the zero one weighting (binary decision) in the selection of APs, the concept here is to reduce the dimensions by combining APs. In other words, the adopted method is information reorganization, rather than AP selection. As shown in Equation (2), the principal components ${\left[y_{1},{\;y}_{2},\;K,{\;y}_{U}\right]}^{T}$ are produced by a transformation with real numbers. With appropriate weightings, the information transmitted into Y from X can be maximized.

(2) $${\left[\begin{array}{c} y_{1}\\ \begin{array}{c} M\\ \begin{array}{c} M\\ y_{U} \end{array} \end{array} \end{array}\right]}_{U\times 1}={\left[\begin{array}{ccc} a_{11} & a_{12} & \begin{array}{cc} L & a_{1M} \end{array}\\ a_{21} & a_{22} & \begin{array}{cc} L & a_{2M} \end{array}\\ \begin{array}{c} M\\ a_{U1} \end{array} & \begin{array}{c} M\\ a_{U2} \end{array} & \begin{array}{c} \begin{array}{cc} M & M \end{array}\\ \begin{array}{cc} L & a_{UM} \end{array} \end{array} \end{array}\right]}_{U\times M}{\left[\begin{array}{c} x_{1}\\ M\\ \begin{array}{c} M\\ x_{M} \end{array} \end{array}\right]}_{M\times 1},$$

Subsequently, the computational complexity addressed by machine learning approaches is optimized by reducing the feature space by finding its principal components. Since each dimension of a location fingerprint corresponds to RSSI measurements from a particular AP in the region, the dimension reduction technique (PCA) can be used to reduce fingerprints dimensions from M to U, where U<M.

**Figure 1:**
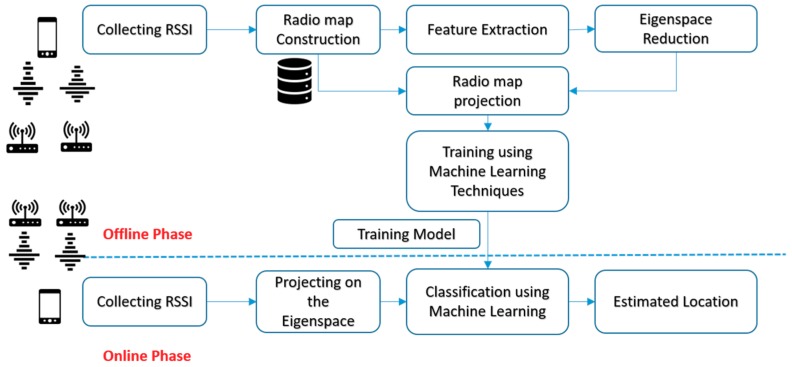
Proposed System Architecture.

The proposed method is divided into two stages as shown in Figure 1. The first one is the offline stage where the radio map is constructed, and the second one is the online stage where measured RSSI data is collected. In the offline stage, the RSSI fingerprints are collected from numerous grid points and used to build the radio map. The radio map was built by saving the RSSI fingerprint at each grid points. Two-dimensional coordinates define each grid points. In order to reduce the high correlation between the grid points and its adjacent point, the PCA is used to generate uncorrelated space. The main two advantages of proposing the PCA algorithm in the offline stage are: (1) The PCA extracts the vital information from the predefined radio map; (2) the PCA reduces the multivariate data matrix without losing much information in which several inter-correlated quantitative dependent variables describe observations.

The PCA algorithm deals with the high dimensionality predefined radio map by linearly combining the features into an uncorrelated space (i.e., Eigenspace) using training covariance matrix of the predefined radio map of size N × N. The radio map matrix is projected into the uncorrelated space in the direction of the most considerable variance. The selection of PCs is based on the highest variance (i.e., eigenvalue). The eigenvalue represents the information context of each PC.

The PCA eigenspace is created based on a set of M-RSSI readings per location of vector $x_{i}$ of size M × 1 as a column vector, where M is smaller than N. This Eigenspace is characterized by the corresponding mean, where N is the number of training samples:

(3) $$\bar{x}=\overset{N}{\underset{i=1}{\sum }}\frac{x_{i}}{N},$$

(4) $$C_{r}=\overset{N}{\underset{i=1}{\sum }}\left(x_{i}-\bar{x}\right){\left(x_{i}-\bar{x}\right)}^{T}=X_{i}X_{i}^{T},$$

where $C_{r}$ is the covariance matrix, computed from the set of N samples.

Let the eigenvalues $\left\{\lambda_{1},{\;\lambda }_{2},\ldots ,{\;\lambda }_{N}\right\}$ of $C_{r}$ that are arranged in descending order with corresponding normalized eigenvectors $\left\{V_{1},{\;V}_{2},\;\ldots ,{\;V}_{N}\right\}$ as follows:

(5) $$C_{r}V_{i}=\lambda_{i}V_{i}$$

(6) $$\lambda_{1}\geq \;\lambda_{2}\;\geq \ldots \;\geq \lambda_{N}$$

where eigenvectors $\left\{V_{1},{\;V}_{2},\;\ldots ,{\;V}_{N}\right\}$ are geometrically orthonormal and statistically uncorrelated.

Before projecting the radio map on the eigenspace, it will be reduced by selecting the minimum number of PCs. Choosing the minimum number of PCs will eliminate the duplication and decrease the noise of the data. The minimum number of PCs in this research is determined based on using two methodologies, where (1) the importance of information context of each PC is evaluated through the value of the eigenvalue, which represent the variance of the data around the PC. As the eigenvalue of the corresponding PC increases, it tends to contain more valuable information; (2) the rule of selecting the number of PCs is based on the cumulative percentage of eigenvalues.

The selection of the number of principal components is based on the cumulative percentage of eigenvalues using the rule in Equation (7) as follows:

(7) $$\left(\overset{U}{\underset{i=1}{\sum }}\lambda_{i}\right)/\left(\overset{N}{\underset{i=1}{\sum }}\lambda_{i}\right)>\eta$$

where $\left\{\lambda_{1},{\;\lambda }_{2},\;\ldots ,\lambda_{N}\right\}$ are the eigenvalues, $(\overset{U}{\underset{i=1}{\sum }}\lambda_{i})/\left(\overset{N}{\underset{i=1}{\sum }}\lambda_{i}\right)$ which represent the percentage which retains the information with its’ information context and η is the cut-off threshold.

The uncorrelated new radio map $Z_{i}$ is calculated by projecting $X_{i}$ on the reduced eigenspace W, which represents a linear combination of the eigenvectors, where $Z_{i}$ will represent the new radio map.

(8) W=X×V

(9) $$Z_{i}=W^{T}\times X_{i}\;\forall i$$

Several classifiers will be trained using the new radio map $Z_{i}$, namely, the K-Nearest Neighbor (KNN), the support vector machine (SVM), and the decision trees (DT), which will be introduced briefly in the following sections, generating the training model, which was reduced after selecting PCs from eigenspace where the essential information contexts are extracted from the radio map.

In the online stage, the tested RSSI fingerprints that are collected are then projected on the eigenspace W, which was processed at the offline stage. The projected values from the tested RSSI fingerprints are compared with the trained model to find the best match between them using the proposed classifiers. The physical location of the model, which has the best match in the new radio map, will be labelled as the estimated location.

### 3.1. K-Nearest Neighbor (KNN)

K-nearest neighbor is a method that tries to perform classification by calculating the distance between features [9,17]. The values of RSSI fingerprints depend on the physical distance between APs used in radio map and mobile phone. The KNN algorithm considers K calibration points. The selection of these points based on selecting the closest K points in the feature space to approximate the position of the user. In the literature [9], it was mentioned that selecting the nearest point is a good indication of closeness in the physical space.

The KNN algorithm starts by calculating the P-norm of M-dimensions RSSI vector $\left(x_{i}\right)$, where $x_{i}$ belongs to the fingerprint radio map $R^{N}$. The KNN algorithm calculates the distance between the measured $\bar{y}$ and the RSSI of the vector of the radio map $\bar{x_{i}}$.

### 3.2. Support Vector Machines (SVMs)

Support Vector Machines [14,18] are a powerful technique used for classification and data regression. They are used as a nonparametric supervised classifier for pattern recognition problems. SVMs are used in the localization system by training the support vectors on the radio map that consist of grid points. SVMs analyze the relationship between the trained fingerprints and their grid points by considering each grid point as a class. The tested RSSI fingerprints are taken as an input to SVM that predict the class to which the tested belongs (i.e., grid point). This technique can be generalized to classify between more than two classes for N training data $\left(x_{i},{\;y}_{i}\right)$.

Before any classification, the RSSI fingerprint vectors are mapped into higher dimensional space using kernel functions. The SVM kernel functions K(. , .) is the dot product of two feature vectors $x_{i}$ and $x_{j}$ in some expanded feature space, and there are several kernels which are proposed in the literature [14]. The four basic kernels used in this paper are shown in Reference [14] as follows: Linear, polynomial, sigmoid, and radial basis functions (RBF). The grids in the radio map turn the indoor localization problem to multiclass classification problems which are combined in two classes and which are labelled as binary (i.e., pairwise). This can be applied in two ways one-against-all and one-against-one [18]. Therefore, given a fingerprint that is not present in the training dataset, the classifier should provide the label of the grid where it was measured. The one-versus-all approach is constructed by dividing the multiclass problem into a group of pair classification problems. The one-versus-all technique is advantageous from a computational standpoint, in that it only requires many classifiers equal to the number of classes. The one-versus-one decomposes the multiclass problem into the set of all possible one-versus-one problems. Thus, for an n-class problem, n (n−1)/2 classifiers must be designed to create a disadvantage of the increase in the number of classifiers required as compared to one-versus-all.

### 3.3. Decision Tree

Decision trees (DTs) [19] are nonparametric supervised learning method used for regression and classification. The DTs is an algorithm based on a binary decision tree that is constructed from the training data set. The algorithm starts by learning simple decision rules inferred from the data features. The algorithm stops when there is a real decision and uncertainty is inefficient. A real decision means that each node’s data subset contains one and only one target location.

The node’s data subset splitting rule is the Gini diversity index [19]:

(10) Gini(t)=∑i=1Np(it)[1−p(it)]=1−∑i=1N[p(it)]2,

where p(it) is the probability of class *i*^th^ occurring at a certain node in the tree.

### 3.4. Random Forest

Random forest (RF) [19] is a classifier in which many decision trees are generated. The RF chooses the tree which has the highest votes after their classification results. The most occurring class number in the output of the decision trees is the final output of the RF classifier. The RF classifier allows the estimation of the importance of each feature in those classification results. A recursive process in which the input dataset is composed of smaller subsets allows the training of each decision tree. This process continues until all the tree nodes reach similar output targets. The random forest classifier takes weights based on the input as a parameter that resembles the number of decision trees. Those weights will be formed in the collaborative forest classifier without the conventional tree pruning process.

## 4. Experimental Work

In this paper, a mobile phone—Galaxy Note 10.1—is used in our experiments, which is used to collect real data utilizing a developed android application. The developed application was used to scan all the channels and the reachable APs of the selected environments. The selected approach in collecting data was based on a server processing base [20] which is used in both training and testing stages, therefore, the mobile device reports its RSSI measurements to the server, and the radio map is constructed. The collected RSSI measurements are uploaded to the server using the UDP protocol, which is utilized in the android application to connect to the server.

**Figure 2:**
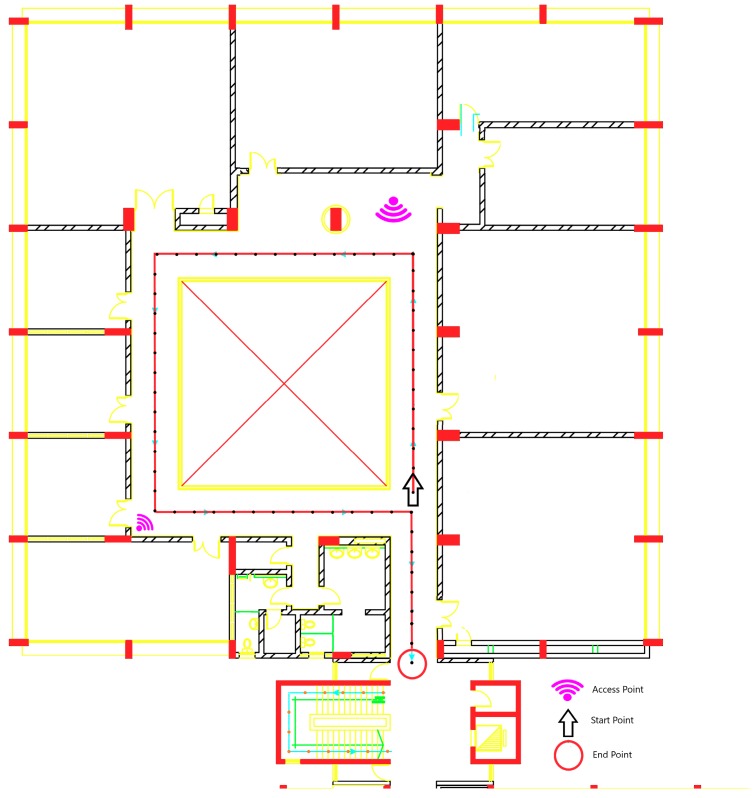
The layout of the residence where the second experiment was conducted.

In Figure 2, the layout represents a part of the first floor plan of the International Transport and Logistics College building at the Arab Academy for Science, Technology, and Maritime Transport (AASTMT) in Alexandria, Egypt. The floor contains mainly offices, conference rooms, computer labs, and bathrooms. There is a random interfering motion of students and employees added to the indoor environment. The experiment is handled over 2D area of length 30 m and width of 32 m, the overall is 960 m^2^, which is considered a larger area for the application than the reported database in Reference [21] with an area of 600 m^2^.

The data were collected during February 2018. The red path, shown in Figure 2, is the actual path in the experiment, which consists of 70 grid points that are colored with black points. Each grid point is separated with 1 m of length. There were six available 802.11 Wi-Fi APs distributed in the floor. All of them are from the same vendor and the same model, which is Cisco Aironet 1600 Series Access Point (Model: Cisco AIR-SAP1602I-I-K9) [22]. The APs are operating in Dual-band 2.4-GHz and 5-GHz 802.11n with Multiple Input Multiple Output (MIMO) radio system. The APs are configured to work with Multiple Service Set Identifier (SSID) with Multiple Virtual Local Area Networks (VLANs). The reachable APs from the previously mentioned features are around 161 SSID with different MAC Address (Media Access Control Address). The training sets are collected in separated days of five training sets that are collected to form the radio map. Each training set took 24 min, where each grid point takes 20 s with nine samples as RSSI readings.

For the offline stage, 20 s RSSI measurements were collected at each grip point’s location. In order to build the radio map, five training sets were collected by walking in a regular walk within one week. For the testing stage, the trajectory of the experiment was a regular walk through all 70 grids points, as shown in red color in Figure 2, for the same duration for the trajectory.

## 5. Results and Analysis

This section will start by discussing the important factors of evaluating the parameters and methodologies that will be used in the deterministic and machine learning approach. Then, a statistical analysis for data upon each information that the PC holds will be introduced. After this, the mean error is calculated with the chosen parameters relative to the selected index of PCs. Finally, applying all the chosen parameters, the performances of the deterministic and machine learning approach will be compared.

The used metrics to compare between the performances of the classifiers are the mean error and Cumulative Distributed Function (CDF) of the distance error to measure the precision of the used methods after and before the dimension reduction. Also, the reduction in the size of the radio map is calculated. These metrics are evaluated using an experiment for a regular walk through all grid points in the radio map.

### 5.1. Analysis of PCs

**Figure 3:**
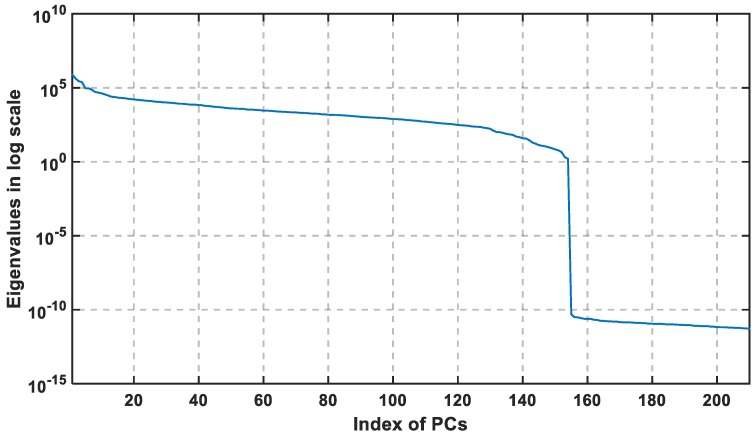
First 210 Eigenvalues (Log Scaled) of Large-Scale Experiment.

**Figure 4:**
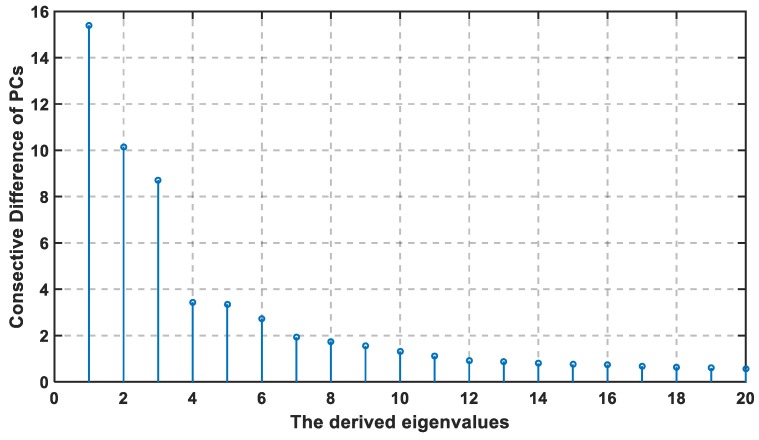
Derived Eigenvalues in Large-Scale Experiment.

For the dataset collected in this paper, the training set is constructed of 70 grid points and the collected training sets are at different conditions, where the total training samples are 350 samples. Each sample is constructed of 161 features (APs). The constructed covariance matrix will have a size of 350 × 350 that will generate 350 PCs. In Figure 3, the first method discusses the importance of information in the data by choosing the PC with the largest eigenvalue that indicates the corresponding variance of PC. The first non-zero elements are the first 210 PCs, which mean that all the valuable information are found in them. Therefore, the principal component analysis has made here a short description from 161 feature to 154, but also indicates the significance of the first 20 PCs. In Figure 4, the logarithmic scale is plotted for all PCs, and the highest eigenvalues are concentrated in the first 20 principal components, which means that the valuable information is concentrated in the first 20 PCs that decrease the region of searching.

The selected threshold value is adjusted to be 85% of the total information. The cumulative percentage is calculated from the 10th PC to 18th, where they are arranged, respectively, at 78%, 79.33%, 80.45%, 81.36%, 83.24%, 83.79%, 83.79%, 84.52%, and 85.19%. The selected PC is the integer number where the cumulative percentage exceeds the cut-off threshold. Therefore, the selected PC with the previous method is at U equal to 18 or greater.

**Figure 5:**
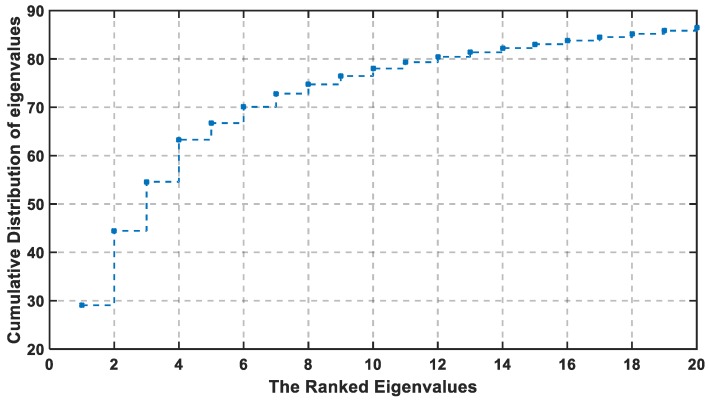
Cumulative Distribution of the Eigenvalues Versus the Number of PCs U in Large-Scale Experiment.

The consecutive difference of eigenvalues are reported in Figure 4 with descending order, and its cumulative distribution is shown in Figure 5. According to both figures, the first four eigenvalues are the largest in value and difference, while the last fourteen consecutive difference is closely equal to each other. Hence, the slope in Figure 5 is the sharpest between 1 and 4, then decreases from 5 to 20. At U ≥ 18, the slope in Figure 5 becomes much lower, and the last eight dimensions (13 to 20) account for only 7% of the information. If the cut-off threshold is set as 85%, then the required U is 18, so the chosen percentage is exceeded, where the remaining 15% can be the noise and the duplication in the data. 

The RSSI value is an environment dependent. Thus, the only case that makes the principal component not the optimum approach to extract the valuable information and exclude the added noise, is if a highly nonlinear effect exists between RSSI measurements.

### 5.2. Method with Machine Learning Approach

**Figure 6:**
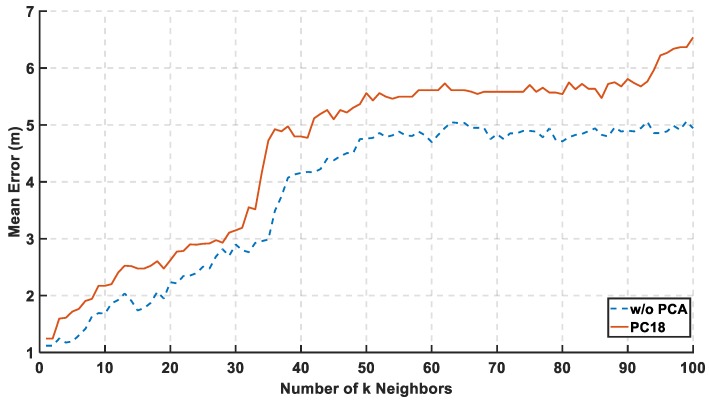
Mean Error is Calculated Relative to the Number of k Neighbors using the 18th Principal Component.

In Figure 6, the mean error is calculated for the 18th PCs. The general propose from this figure is choosing the k for the lowest complexity and mean error. Thus, the maximum difference between using 18 and 161 features is 1.6 m. The figure shows that the mean error is always increasing with k because the real grid points are highly separated in the physical space. The best value for k is chosen with the lowest calculation complexity and mean error is at (k = 1), where other authors [9] observed the same value in a large-scale environment. Varying the number of neighbors k in KNN method implies better results in small values of k because, for large values of k, the points are far away from the exact location which corrupts the estimate location.

**Figure 7:**
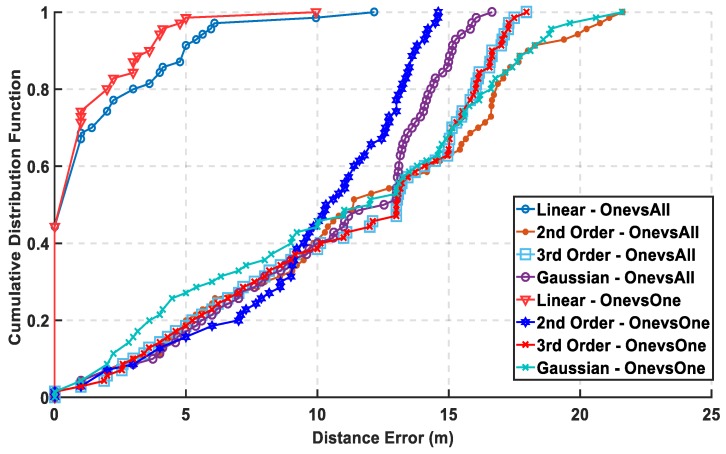
Cumulative Distribution Function using One-Versus-One and One-Versus-All Approaches for Support Vector Machine (SVM) without Principal Component Analysis (PCA).

**Figure 8:**
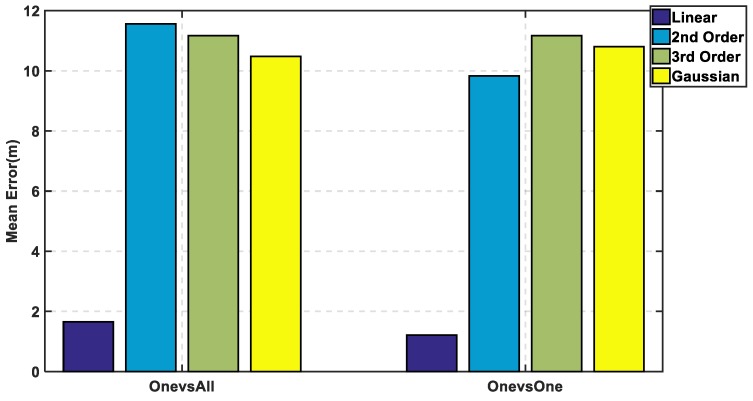
Mean Error using One-Versus-All and One-Versus-One Approaches for SVM without PCA.

In Figure 7, the max cumulative error is calculated for the two approaches showing the lowest accumulated error using the linear kernel by the one-versus-one approach, while there is an overlapping between the third order for both approaches which means using different complex kernels will not solve this classification problem. The linear kernel for both approaches reaches 100% of CDF faster than the other kernels indicating for high precision. As for the mean error in Figure 8, it is calculated in 8 for both approaches and the lowest mean error is also the linear kernel. Both metrics indicate the performance of the classifiers to approve that the linear kernel with one-versus-one is the chosen kernel to reveal a mean error of 1.22 m, while the cumulated error at 5 m is 98.6% without PCA.

**Figure 9:**
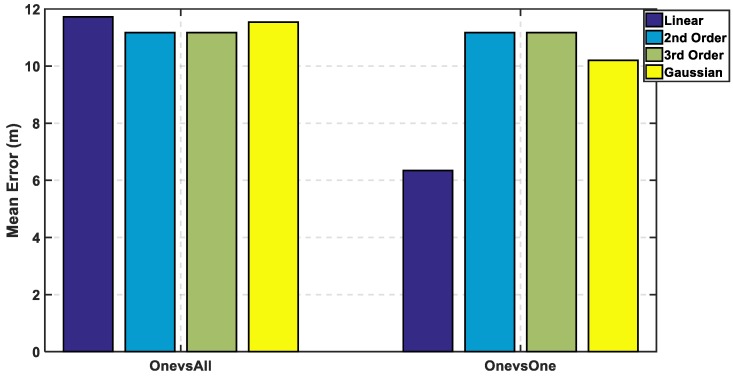
Mean Error for One-Versus-One and One-Versus-All Approaches for SVM at the 8th Principal Component.

**Figure 10:**
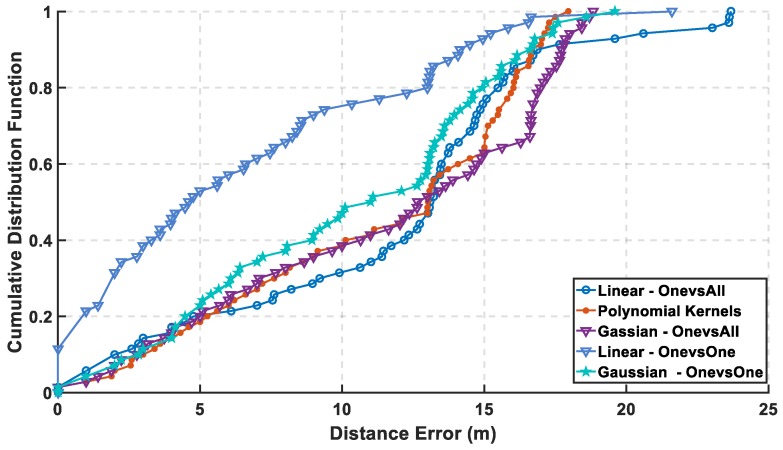
Cumulative Distribution Function using One-Versus-One and One-Versus-All Approaches for SVM using the 18th PC.

In Figure 9, the mean error is calculated for both approaches, where the linear kernel using one-versus-one approach has the lowest mean error. The mean error is increased from 1.22 m to 6.3 m, which is a difference around 5 m that reveals a bad performance but is fair after using 18 out of 161 features, which reflects in decreasing the computational complexity. In Figure 10, the cumulative error is calculated for both approaches, where it reaches a cumulated error at 10 m of 75.7% for linear kernel using the one-versus-one approach in contrast to before reduction of the radio map to reach 100% at the same level in Figure 7. The other kernels are lower than 48% in both the approaches.

**Figure 11:**
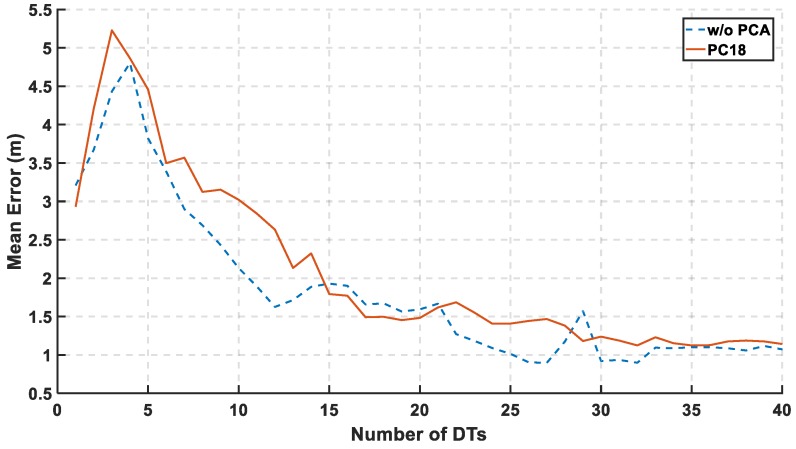
Mean Error Relative to the Number of Decision Trees (DTs) using 18th PCs.

In Figure 11, the mean error is calculated relative to the number of DTs. The used range of the decision tree is from 1 to 40. The significant decrease in the mean error started after third DTs to reach the lowest mean at 32th DTs.

### 5.3. Performance Evaluation

The mean and accumulated error is evaluated at the different number of PCs to confirm that the 18th PCs have acceptable values to reduce the computational complexity and also decrease the size of the radio map. The size of the radio map is reduced to be 11.8% of the original radio map. The latter calculations are compared in the next analysis to show the effect of the reduction in the computational complexity that is satisfied as a result of using of a new projection of the radio map using the selected PCs, where the CDF and mean error are also used to compare between the performances of the classifiers.

**Figure 12:**
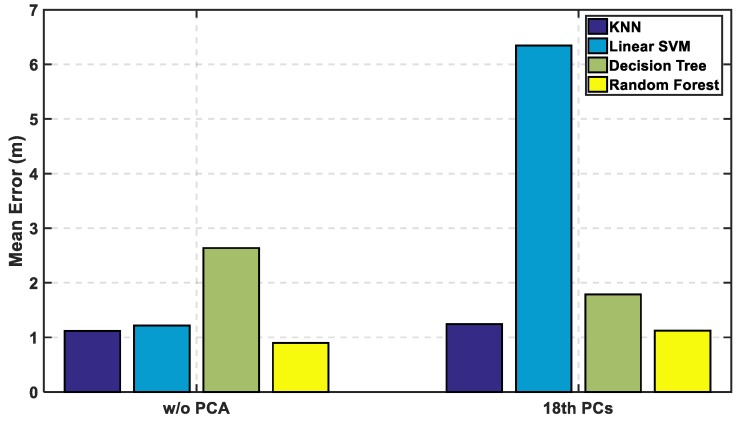
Mean Error Versus the Machine Learning Approach with and without PCA.

**Figure 13:**
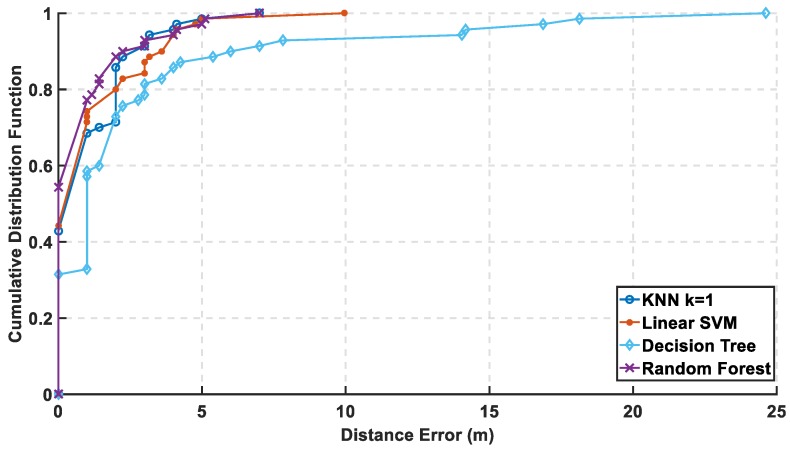
Cumulative Distribution Function (CDF) Error of the Classifier without PCA.

**Figure 14:**
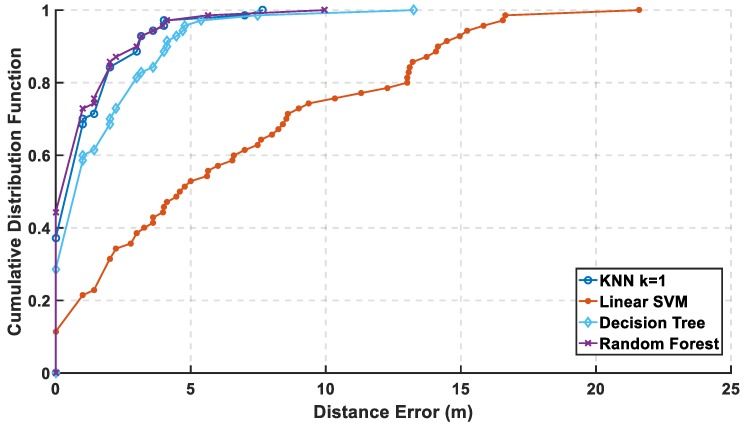
CDF Error with PCA with 18th PCs.

In Figure 12, the mean error is calculated with and without PCA, and it is observed that KNN and random forest are enhanced saving their level of accuracy, while the decision tree performance shown is reasonably improved. As for SVM, it shows an apparent lousy performance after the reduction of the radio map. In Figure 13 and Figure 14, the performance of the classifiers with and without using PCA regarding CDF error is presented. The zero-distance error in the CDF is almost the same percentage with and without PCA, which indicates high performance and accuracy using the reduced radio map, reaching 32% of the experiment. Table 1 shows the numerical results of CDF value at 3 m with and without PCA, which provides almost the same accuracy in other classifiers but not with linear SVM. Table 2 shows the error distance equivalent to the percentage of cumulated error at 100%. The precision is identified according to the minimum error distance at 100% of cumulated error, where the KNN has the same accuracy, while the linear SVM has lousy performance. The decision tree is highly enhanced, but the random forest is still the best.

## 6. Conclusions

One of the significant challenges at present is the unavailability of satellite-based navigation and positioning systems in all environments. For example, the common satellite-based navigation system cannot work inside buildings due to signal blockage and dense multipath problems. Several studies in the literature already investigated some of the aspects associated with building a WLAN-based indoor location fingerprinting system using the machine learning approach. This approach is based on finding the perfect match between the user fingerprint and predefined set of grid points on the radio map. This paper proposed an indoor positioning system technique based on principal component analysis that offers a more efficient methodology to utilize information from all APs and maintain the balance between the position accuracy and computational cost, especially for the large-scale indoor environments. To evaluate the performance of the proposed technique, data was collected from a realistic large-scale indoor WLAN environment with an area of 960 m^2^. The maximum reachable access points were over 161 APs. The proposed technique shows significant reduction of computation cost using several machine learning techniques. The results show that the radio map is reduced by 11.18 percent and the data feature complexity in each location is decreased into 18 features instead of 161 features after discarding the worthless APs. A possible future extension is applying the proposed method on a multi-floor radio map.

## Figures and Tables

**Table 1:** The CDF Error within 3 m.

Classifier	Without PCA	With PCA
KNN	98.5%	98.5%
Linear SVM	98.5%	53%
Decision Tree	88.57%	98.5%
Random Forest	98.5%	98.5%

**Table 2:** The CDF Error at 100%.

Classifier	Without PCA	With PCA
KNN (K = 1)	7 m	7.67 m
Linear SVM	9.95 m	21.6 m
Decision Tree	24.62 m	13.26 m
Random Forest	7 m	9.95 m
